# Correction: Tissue-specific and *cis*-regulatory changes underlie parallel, adaptive gene expression evolution in house mice

**DOI:** 10.1371/journal.pgen.1011213

**Published:** 2024-03-27

**Authors:** The PLOS Genetics Staff

## Notice of republication

This article was republished on March 7, 2024, to correct references 18–47 in the Reference list and their linked in-text citations. The publisher apologizes for the errors. Please download this article again to view the correct version. The originally published, uncorrected article and the republished, corrected articles are provided here for reference.

## Supporting information

S1 File Originally published, uncorrected article. (PDF)

S2 File Republished, corrected article. (PDF)
