# Supplementary material for: Tissue-specific and cis-regulatory changes underlie parallel, adaptive gene expression evolution in house mice
Source: PLoS Genet. 2024 Feb 2;20(2):e1010892. doi: 10.1371/journal.pgen.1010892 (PMC10866503; doi:10.1371/journal.pgen.1010892)
Supplement: S1 Appendix — (DOCX) [file pgen.1010892.s001.docx]

**S1 Appendix: Supplementary results of shared vs. unique DEG patterns and comparisons of exome and transcriptome variation.**

**1. Unique and shared differential expression analyses**

**Methods:**

To understand patterns of shared and unique differentially expressed genes (DEGs) between the SARA-MANA and EDMA-MANA comparisons, we conducted gene regulatory category, tissue specificity enrichment, and increased body size enrichment analyses using unique and shared DEGs. After defining unique and shared gene sets for each tissue and each comparison, regulatory categories were determined as described in the “*Cis- and trans-regulatory assignment*” section, and enrichment of DEGs among tissue-specific genes and increased body size genes was done analogously to that described in the “*Permutation tests of enrichment*” section. To assess patterns of selection on shared and unique changes we tested for enrichment of shared and unique significant ASE genes analogously to that described in the “*PBS tests for selection*” section. For this analysis we used an outlier cutoff of 5% and focused on the second set of tests (NH/VT focal – Florida – Iran and EDM focal – Arizona – Iran), as outliers identified in these tests are most directly tied to selection acting independently in New York and Alberta house mice.

**Results:**

*Regulatory categorization:* For SARA vs. MANA liver, 558 of the genes tested for ASE were significantly differentially expressed, and 218 were unique, while 340 were shared between the SARA and EDMA comparisons (BAT: 474 differentially expressed, 186 unique, 288 shared). For EDMA vs. MANA liver, 635 of the genes tested for ASE were significantly differentially expressed, 309 were unique and 326 were shared (BAT: 477 differentially expressed, 186 unique, 291 shared). The number of shared genes is not the same between comparisons because EDMA and SARA had separate gene sets that were able to be tested for ASE, and “shared” was defined as also differentially expressed between the parent groups of the other comparison.

We found that more genes were *cis-* as opposed to *trans-*regulated in all DEGs, unique DEGs, and shared DEGs in each comparison and tissue (S2 Table). In liver we found that SARA unique DEGs had a greater proportion of *cis*-regulated genes than all or shared DEGs, while EDMA unique DEGs had a greater proportion of *trans*-regulated genes than all or shared DEGs, perhaps reflecting the greater proportion of *trans*-regulation observed in EDMA more generally. However, this trend was not present in BAT.

*Tissue specificity and increased body size genes:* Similar to our results showing enrichment of significant DEGs in tissue-specific genes when all DEGs were analyzed (Fig 5B, S6B Fig, S7B Fig, S8B Fig), we found that unique and shared DEGs in both tissues and comparisons were also enriched among tissue-specific genes to similar degrees as that seen in all DEGs more generally (p < 0.01, calculated via permutation tests). The one exception was SARA unique DEGs among BAT-specific genes (p = 0.170). For increased body size genes, we found no enrichment of differentially expressed genes among all, shared, or unique DEGs.

*Signals of selection*: For both the SARA and EDMA comparisons, we found that shared significant ASE genes in either tissue were significantly enriched among PBSn1 outliers (p ≤ 0.005), while genes with significant ASE that were unique to either comparison were not (p > 0.05, S3 Table). These results give further support for the substantial parallelism seen among New York and Alberta house mice in their adaptation to cold environments.

**2. Exome and transcriptome comparisons**

**Methods:** MANA and EDMA lab strains were derived from house mice originally collected in Manaus, Brazil and Edmonton Alberta, Canada. Therefore, previously published population level exome data from wild caught Manaus, Brazil (MAN) and Edmonton, Alberta, Canada (EDM) house mice are directly comparable to inbred lab strain expression data for MANA and EDMA, respectively. We do not have population level exome data from Saratoga Springs, NY, the original sampling locality of the SARA lab strain, and use published exome data collected from the New Hampshire / Vermont (NH/VT) border as a proxy. These locations are geographically close and have very similar climates, but to further assess the similarity between SARA and NH/VT as compared to the similarity between EDMA and EDM and MANA and MAN, we defined a shared set of SNPs between lab strain transcriptome data and wild caught exome data to ascertain genetic variation among the six separate groups.

Variant calling methods for transcriptome and exome samples are described in the “*Variant calling from transcriptome data”* and *“PBS tests for selection”* sections, respectively. We first merged 30 exome samples from NH/VT, EDM, and MAN (10 from each group) with 18 transcriptome samples from SARA, EDMA, and MANA (6 from each group). We then removed SNPs that were not present in 90% of individuals and those with a minor allele frequency below 5%. This resulted in a set of 91,884 biallelic SNPs for downstream analyses. We constructed a PCA with the R package SNPRelate [1] and built a neighbor joining tree using vcfR [2], poppr [3], and adegenet [4] in R, and visualized the tree with iTOL [5] (S13 Fig).

**Results:** A PCA of exome and transcriptome samples separates individuals by both data type and sampling locality (S13A Fig), which is likely driven by the lack of genetic variation in inbred strain transcriptome data in comparison to wild caught exome data. We see the same general separation between sampling localities in the exome and transcriptome data, for example, both SARA and NH/VT are pulled to the upper right corner of the plot, with SARA being on the distal edge of the plot. Similar patterns are seen for the other groups. The similarity between SARA and NH/VT grouping as compared to the other groups suggests that the NH/VT exome data can inform interpretation of the SARA expression data in a similar fashion to the way in which the EDM and MAN exome data can inform interpretation of the EDMA and MANA expression data.

Phylogenetic analysis separates samples by sampling locality with NH/VT + SARA, EDM + EDMA, and MAN + MANA all forming monophyletic groups (S13B Fig), with North American groups being more closely related to one another than either is to Brazil.

**References**

1. Zheng X, Levine D, Shen J, Gogarten SM, Laurie C, Weir BS, et al. A high-performance computing toolset for relatedness and principal component analysis of SNP data. 2012;28(24):3326–8.

2. Knaus BJ, Grünwald NJ. vcfr: a package to manipulate and visualize variant call format data in R. Mol Ecol Resour. 2017;17(1):44–53.

3. Kamvar ZN, Tabima JF, Gr̈unwald NJ. Poppr: An R package for genetic analysis of populations with clonal, partially clonal, and/or sexual reproduction. PeerJ. 2014;2:e281.

4. Jombart T, Ahmed I. adegenet 1.3-1: new tools for the analysis of genome-wide SNP data. Bioinformatics. 2011;27(21):3070–1.

5. Letunic I, Bork P. Interactive Tree Of Life (iTOL) v5: an online tool for phylogenetic tree display and annotation. Nucleic Acids Res. 2021;49(W1):W293–6.

| Comparison | Tissue | *cis*-only | *trans*-only | *cis* + *trans* | *cis* x *trans* | Total |
| --- | --- | --- | --- | --- | --- | --- |
| SARAxMANA all DEGs | Liver | 407 (72.9%) | 79 (14.2%) | 42 (7.5%) | 30 (5.4%) | 558 |
| SARAxMANA unique DEGs | Liver | 169 (77.5%) | 25 (11.5%) | 15 (6.9%) | 9 (4.1%) | 218 |
| SARAxMANA shared DEGs | Liver | 238 (70.0%) | 54 (15.9%) | 27 (7.9%) | 21 (6.2%) | 340 |
| SARAxMANA all DEGs | BAT | 338 (71.3%) | 94 (19.8%) | 35 (7.4%) | 7 (1.5%) | 474 |
| SARAxMANA unique DEGs | BAT | 135 (72.6%) | 37 (19.9%) | 12 (6.4%) | 2 (1.1%) | 186 |
| SARAxMANA shared DEGs | BAT | 203 (70.5%) | 57 (19.8%) | 23 (8.0%) | 5 (1.7%) | 288 |
| EDMAxMANA all DEGs | Liver | 343 (54.0%) | 196 (30.9%) | 64 (10.1%) | 32 (5.0%) | 635 |
| EDMAxMANA unique DEGs | Liver | 153 (49.5%) | 110 (35.6%) | 24 (7.8%) | 22 (7.1%) | 309 |
| EDMAxMANA shared DEGs | Liver | 190 (58.3%) | 86 (26.4%) | 40 (12.2%) | 10 (3.1%) | 326 |
| EDMAxMANA all DEGs | BAT | 240 (50.3%) | 152 (31.9%) | 52 (10.9%) | 33 (6.9%) | 477 |
| EDMAxMANA unique DEGs | BAT | 95 (51.1%) | 63 (33.9%) | 15 (8.1%) | 13 (6.9%) | 186 |
| EDMAxMANA shared DEGs | BAT | 145 (49.8%) | 89 (30.6%) | 37 (12.7%) | 20 (6.9%) | 291 |

**S2 Table: Regulation of shared and unique DEGs.**

Gene regulatory distribution of all significantly differentially expressed genes (DEGs), DEGs unique to SARA / EDMA comparisons, and DEGs shared between SARA and EDMA comparisons. Percentages are out of all differentially expressed genes that fell into one of the four regulatory categories: *cis*-only, *trans*-only, *cis* + *trans*, and *cis* x *trans*.

|  | PBS test | Top 5% outliers | Significant ASE | Overlap | p-value |
| --- | --- | --- | --- | --- | --- |
| SARAxMANA  all sig. ASE | NH/VT focal – Florida – Iran | 746 | 1087 | 197 | 0.0025 |
| SARAxMANA unique sig. ASE | NH/VT focal – Florida – Iran | 746 | 666 | 114 | 0.0825 |
| SARAxMANA shared sig. ASE | NH/VT focal – Florida – Iran | 746 | 421 | 83 | 0.005 |
| EDMAxMANA  all sig. ASE | EDM focal – Arizona – Iran | 734 | 1004 | 218 | ~0 |
| EDMAxMANA unique sig. ASE | EDM focal – Arizona – Iran | 734 | 569 | 109 | 0.0623 |
| EDMAxMANA shared sig. ASE | EDM focal – Arizona – Iran | 734 | 435 | 109 | ~0 |

**S3 Table: Signals of selection in shared and unique significant ASE genes.**

Enrichment of all, shared, and unique significant ASE genes in either liver or BAT among top 5% PBSn1 outliers. Only PBSn1 outliers able to be tested for ASE and significant ASE genes represented in the relevant PBSn1 test are used. P-values represent percent of permuted distribution lying outside the observed overlap. Observed overlaps entirely outside the distributed are represented as p = ~0.
