# Supplementary figures and images for: Tissue-specific and cis-regulatory changes underlie parallel, adaptive gene expression evolution in house mice
Source: PLoS Genet. 2024 Feb 2;20(2):e1010892. doi: 10.1371/journal.pgen.1010892 (PMC10866503; doi:10.1371/journal.pgen.1010892)

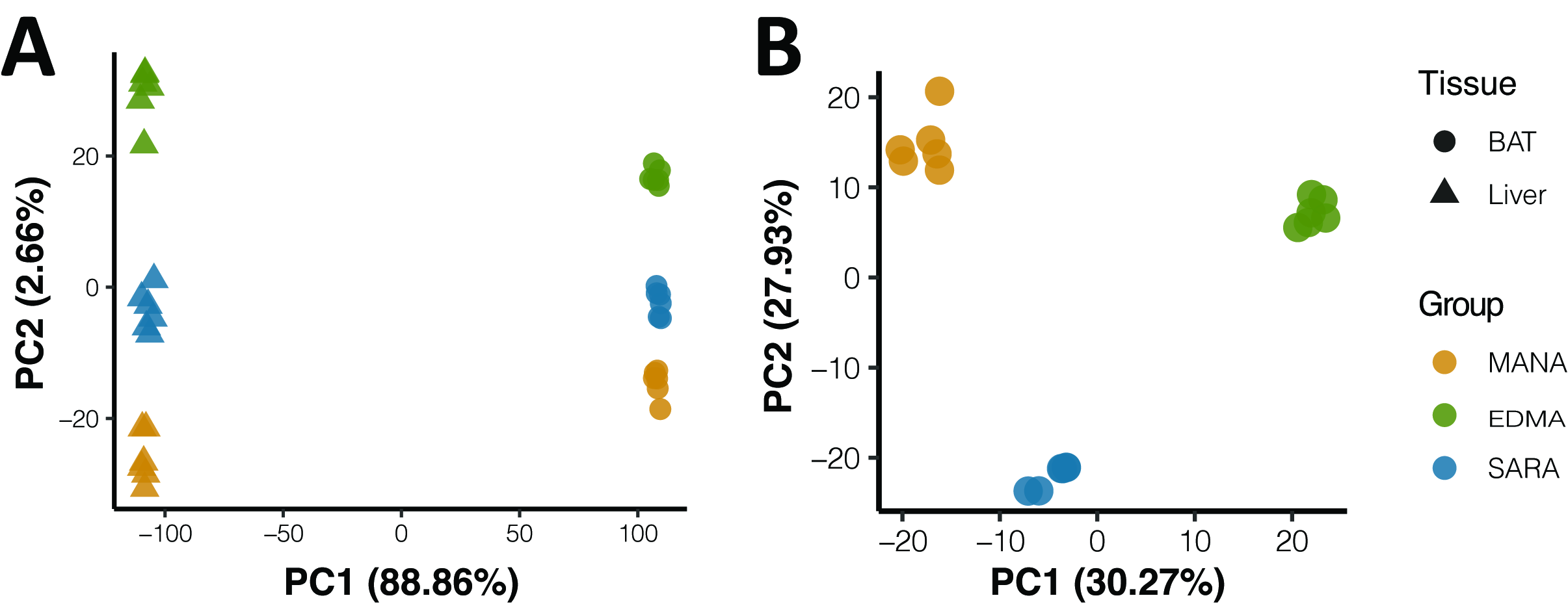

Supplement: S1 Fig — A). Principal component analyses for all RNA-seq data separates samples first on tissue type, and second on original sampling locality. B). Principal component analyses for BAT RNA-seq data separates samples based on original sampling locality. (TIF) [file pgen.1010892.s002.tif]

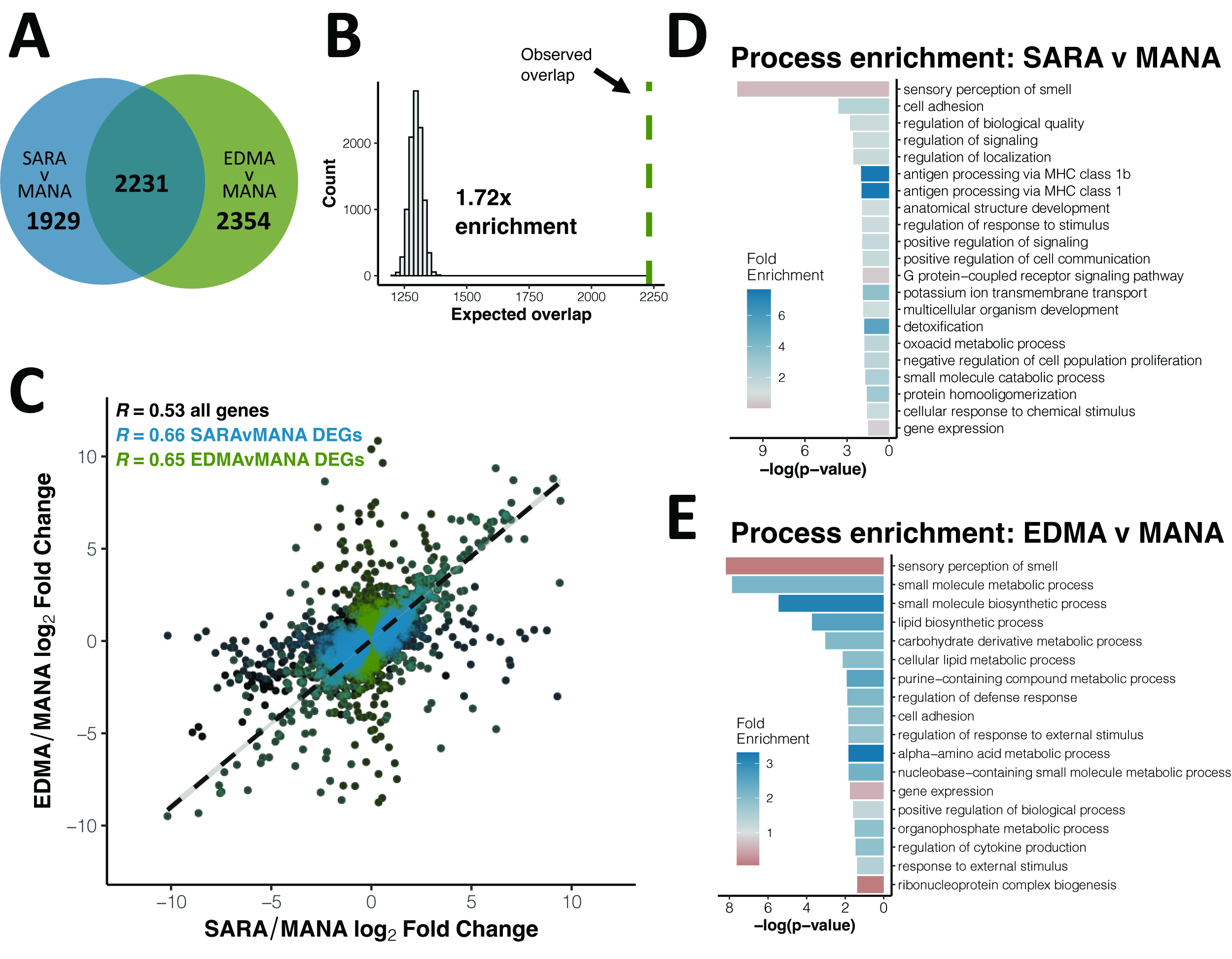

Supplement: S2 Fig — A). Overlap of significantly differentially expressed genes (DEGs) between SARAvMANA and EDMAvMANA comparisons (FDR <5%). B). Expected vs. observed overlap between SARAvMANA and EDMAvMANA DEGs. C). Correlation between SARAvMANA log2 fold change and EDMAvMANA log2 fold change for all genes (black), SARAvMANA DEGs (blue) and EDMAvMANA DEGs (green). Dashed line represents reduced major axis regression for all genes. Correlation coefficients are Spearman’s R. D-E). Gene ontology enrichment for SARAvMANA DEGs (D) and EDMAvMANA DEGs (E). (TIF) [file pgen.1010892.s003.tif]

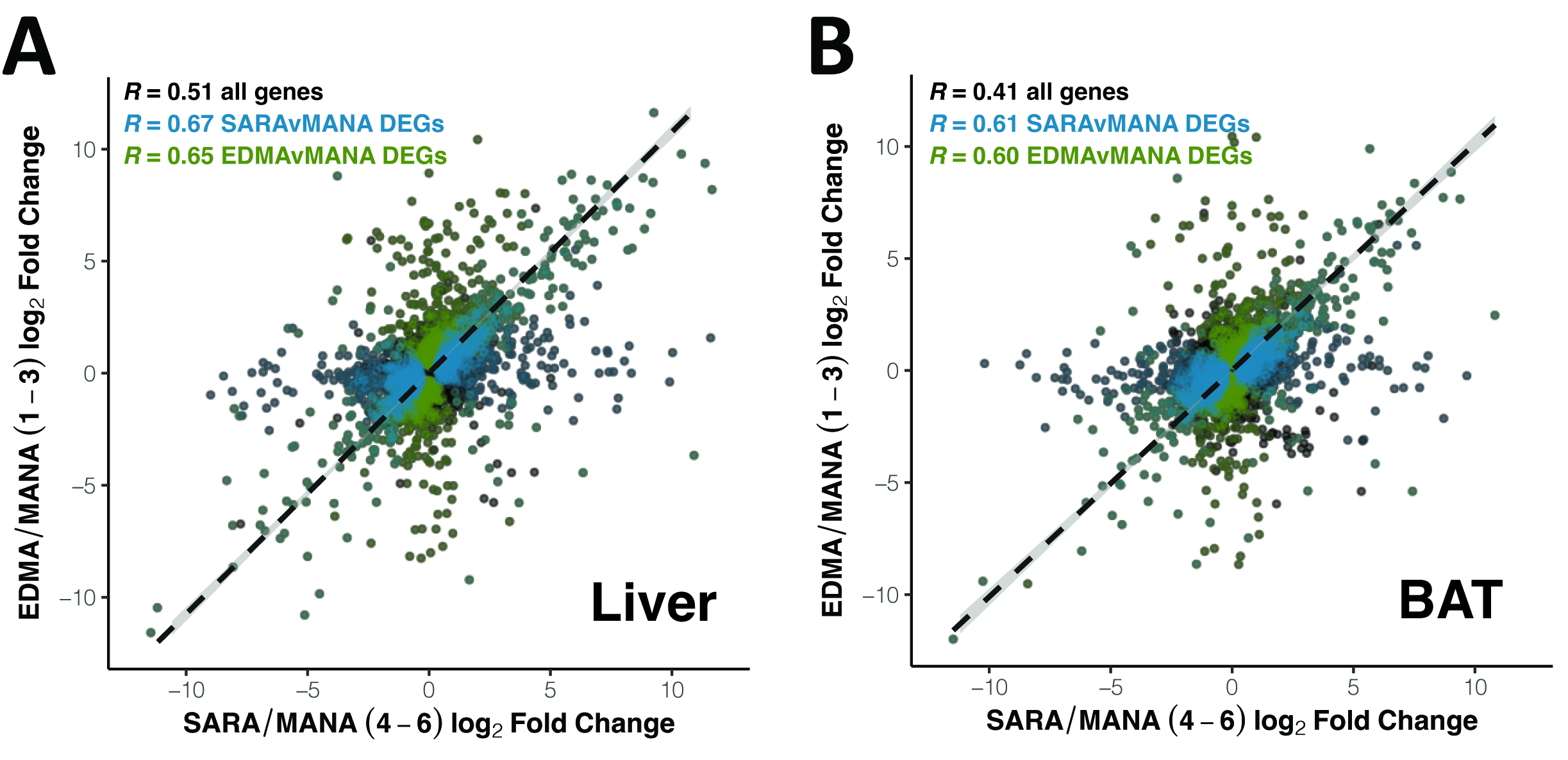

Supplement: S3 Fig — A-B). Correlation between SARAvMANA (MANA samples 4-6) log2 fold change and EDMAvMANA (MANA samples 1-3) log2 fold change in liver (A) and BAT (B). All expressed genes are in black, SARAvMANA DEGs are in blue and EDMAvMANA DEGs are in green. Dashed line represents reduced major axis regression for all genes. Correlation coefficients are Spearman’s R. All significant correlations found using the full MANA data for each comparison are recapitulated. (TIF) [file pgen.1010892.s004.tif]

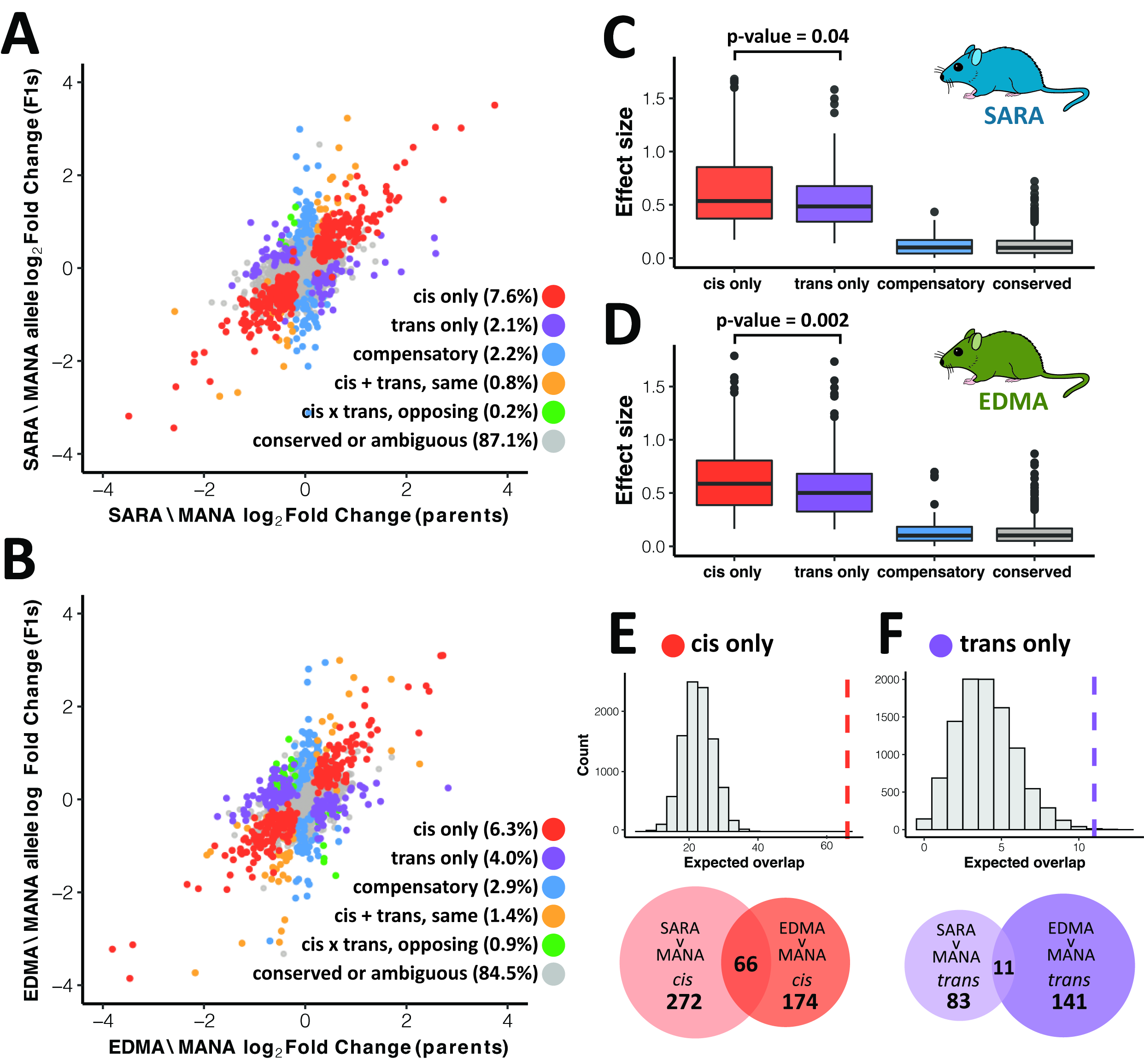

Supplement: S4 Fig — A-B). Correlation between parental differential expression and differential expression between alleles in F1 hybrids for SARAvMANA (A) and EDMAvMANA (B) comparisons. Genes are colored based on regulatory mode (see methods for details). C-D). Effect size as measured as log2 fold change for different regulatory classes in SARAvMANA (C) and EDMAvMANA (D) comparisons. P-values calculated using Welch two sample t-tests. E-F). Expected vs. observed overlap between SARAvMANA and EDMAvMANA cis-regulated (E) and trans-regulated (F) genes. Dashed line indicates observed overlap. Proportion of overlap shown in venn diagrams is significantly higher for cis-regulated genes as compared to trans-regulated genes (Chi-square test of independence, p < 0.0001). (TIF) [file pgen.1010892.s005.tif]

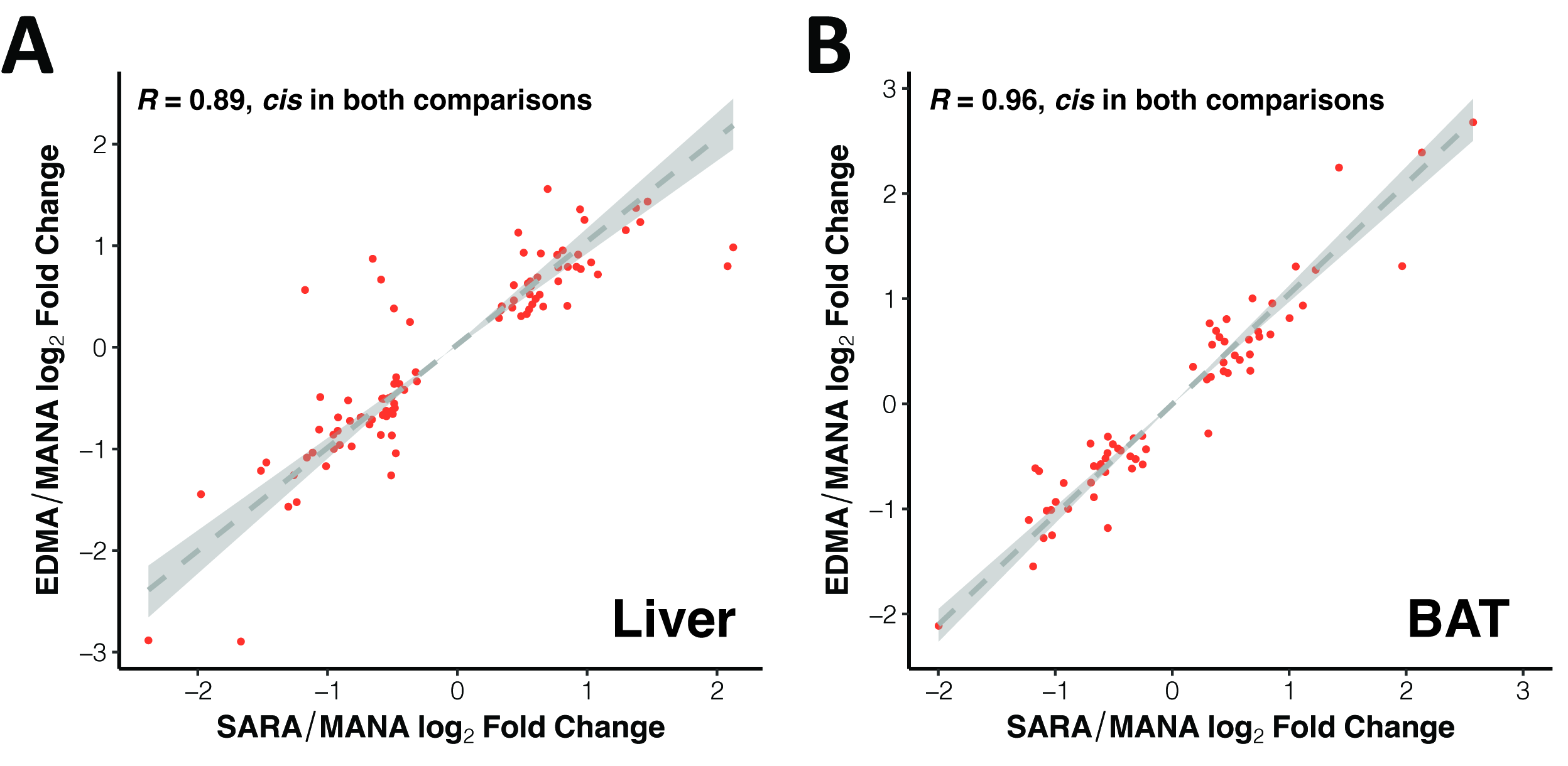

Supplement: S5 Fig — Correlation between SARAvMANA log2 fold change and EDMAvMANA log2 fold change in liver (A) and BAT (B) for genes that are cis-regulated in both comparisons. Dashed line represents reduced major axis regression. Correlation coefficients are Spearman’s R. (TIF) [file pgen.1010892.s006.tif]

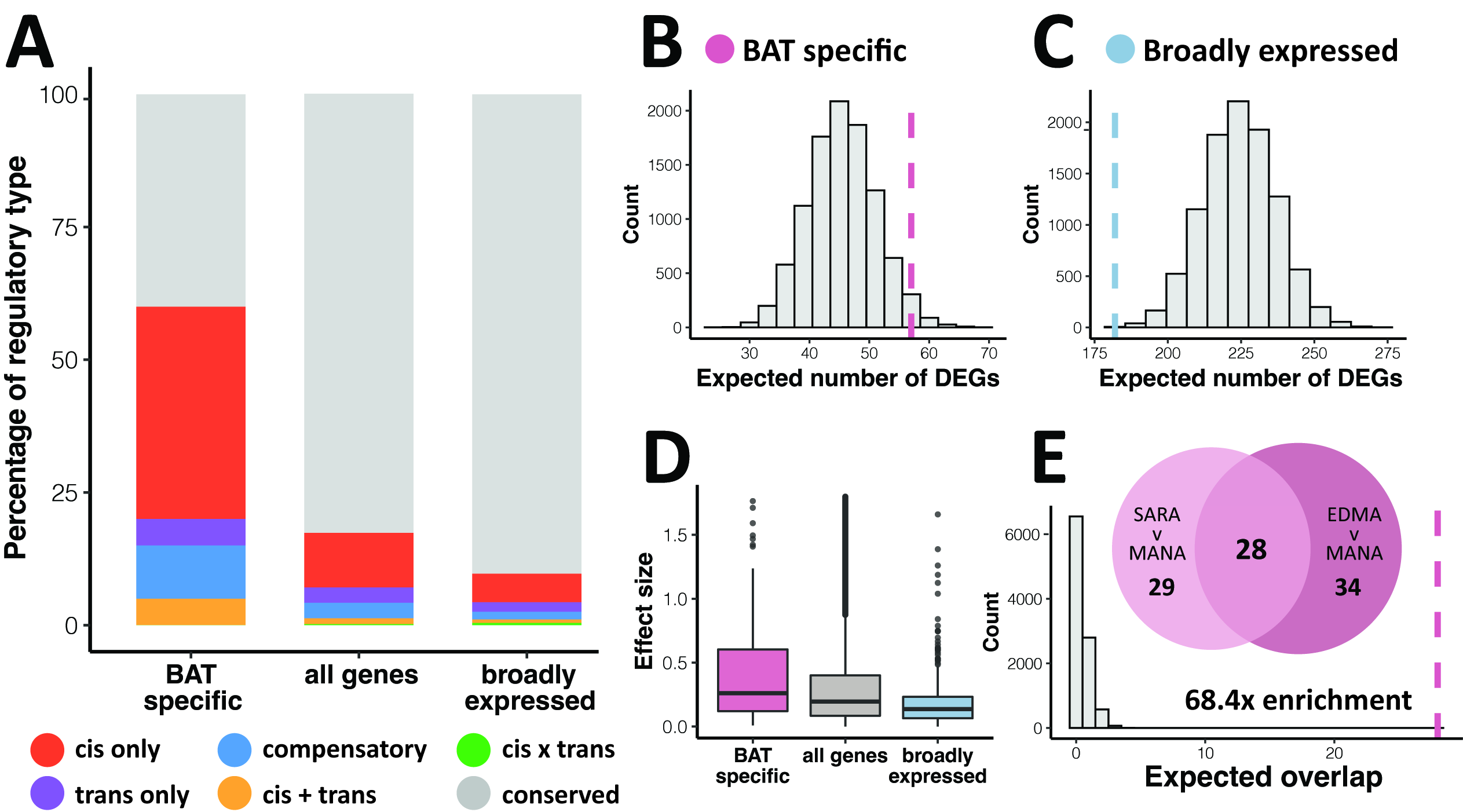

Supplement: S6 Fig — A). Percentage of each regulatory type in BAT-specific, broadly expressed, and all genes. Genes of ambiguous regulatory type are not included. B-C). Expected vs. observed number of significantly differentially expressed genes (DEGs) in BAT-specific (B) and broadly expressed (C) genes. Dashed line indicates observed value. D). Effect size as measured as log2 fold change for BAT-specific, broadly expressed, and all genes. E). Overlap of BAT-specific DEGs between SARAvMANA and EDMAvMANA comparisons (FDR < 5%) and expected vs. observed overlap between SARAvMANA and EDMAvMANA BAT-specific DEGs. Dashed line indicates observed value. (TIF) [file pgen.1010892.s007.tif]

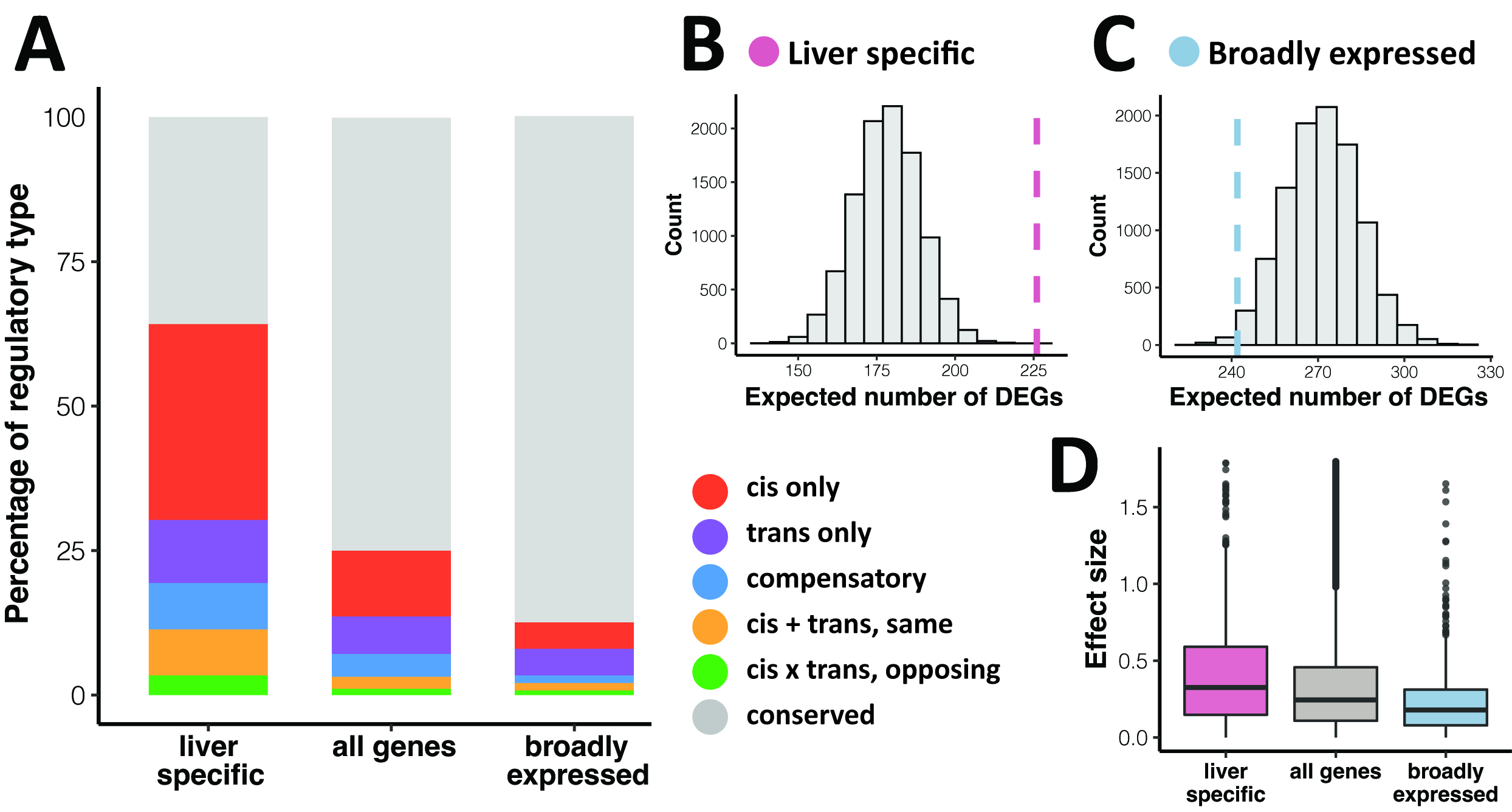

Supplement: S7 Fig — A). Percentage of each regulatory type in liver-specific, broadly expressed, and all genes. Genes of ambiguous regulatory type are not included. B-C). Expected vs. observed number of significantly differentially expressed genes (DEGs) in liver-specific (B) and broadly expressed (C) genes. Dashed line indicates observed value. D). Effect size as measured as log2 fold change for liver-specific, broadly expressed, and all genes. (TIF) [file pgen.1010892.s008.tif]

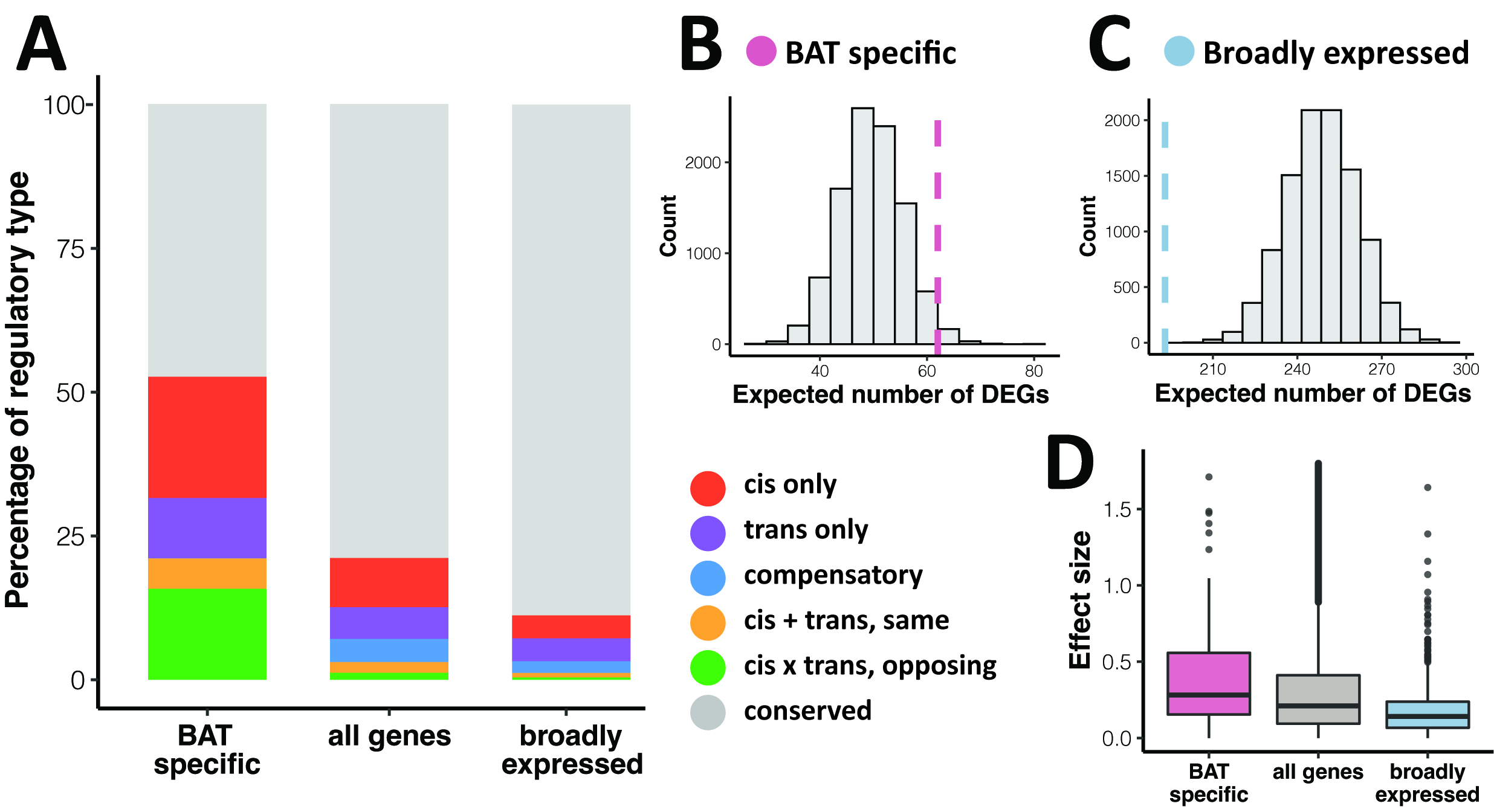

Supplement: S8 Fig — A). Percentage of each regulatory type in BAT-specific, broadly expressed, and all genes. Genes of ambiguous regulatory type are not included. B-C). Expected vs. observed number of significantly differentially expressed genes (DEGs) in BAT-specific (B) and broadly expressed (C) genes. Dashed line indicates observed value. D). Effect size as measured as log2 fold change for BAT-specific, broadly expressed, and all genes. (TIF) [file pgen.1010892.s009.tif]

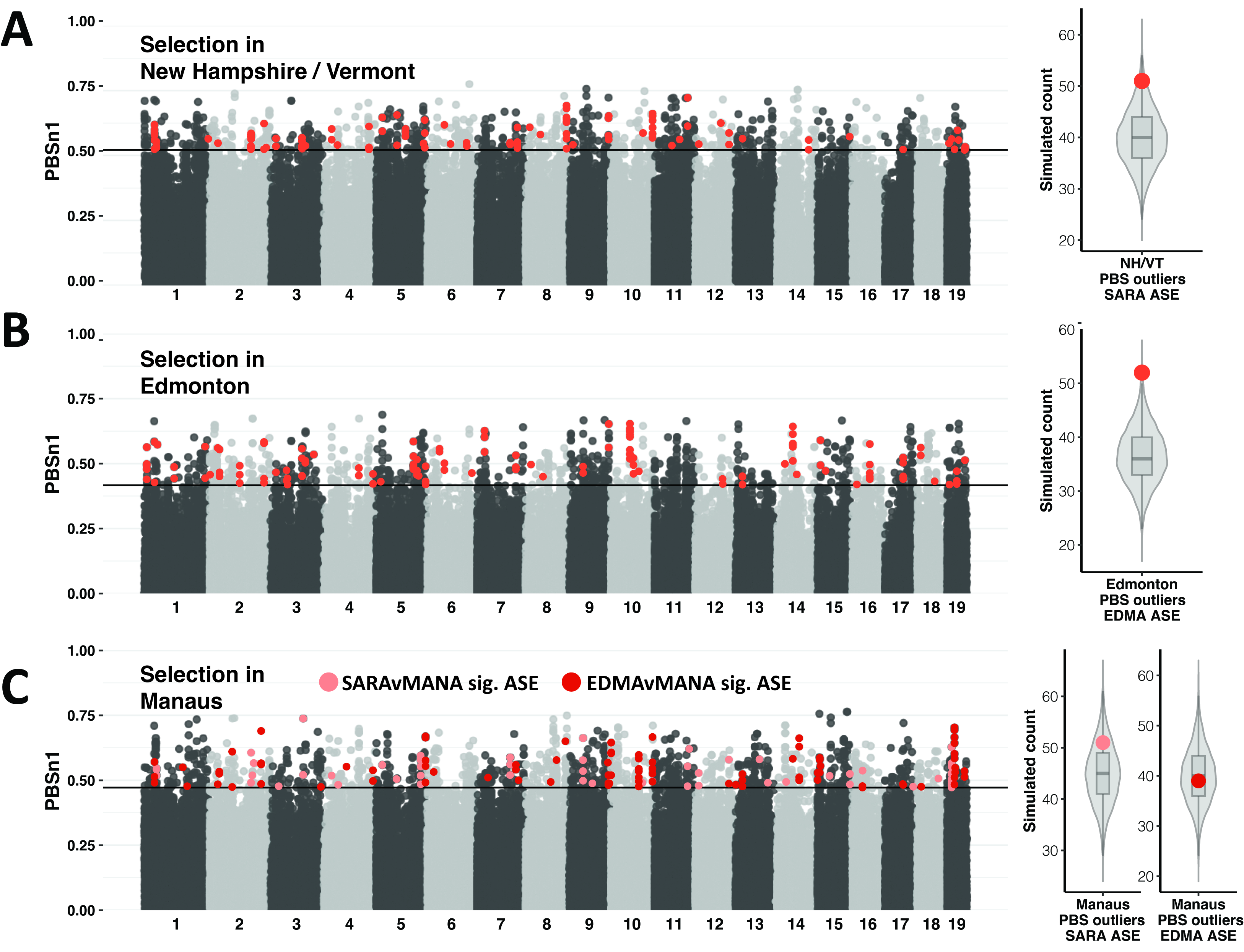

Supplement: S9 Fig — PBSn1 values and expected vs. observed overlaps between genes showing significant ASE and top 1% PBSn1 outliers in the NH/VT-France-Iran test (A) the EDM-France-Iran test (B) and the MAN-France-Iran test (C). Line in Manhattan plots indicates the top 1% cutoff, and colored dots in the permutation test distribution plots indicates the observed overlap. (TIF) [file pgen.1010892.s010.tif]

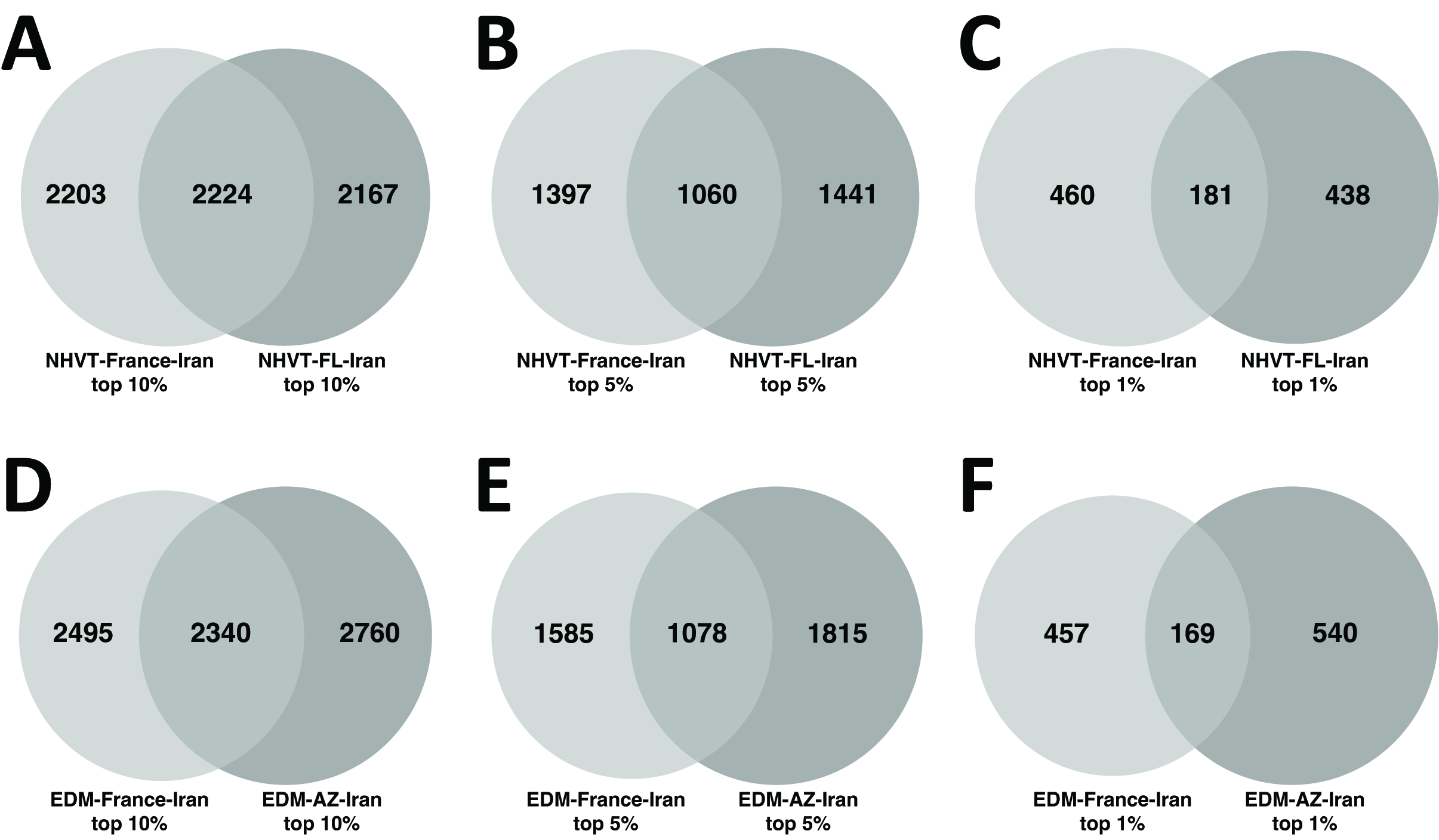

Supplement: S10 Fig — A-C). Overlap of the top 10% (A), 5% (B) and 1% (C) of outlier genes shared between the NH/VT-France-Iran and NH/VT-FL-Iran PBSn1 tests. D-F). Overlap of the top 10% (D), 5% (E) and 1% (F) of outlier genes shared between the EDM-France-Iran and EDM-AZ-Iran PBSn1 tests. (TIF) [file pgen.1010892.s011.tif]

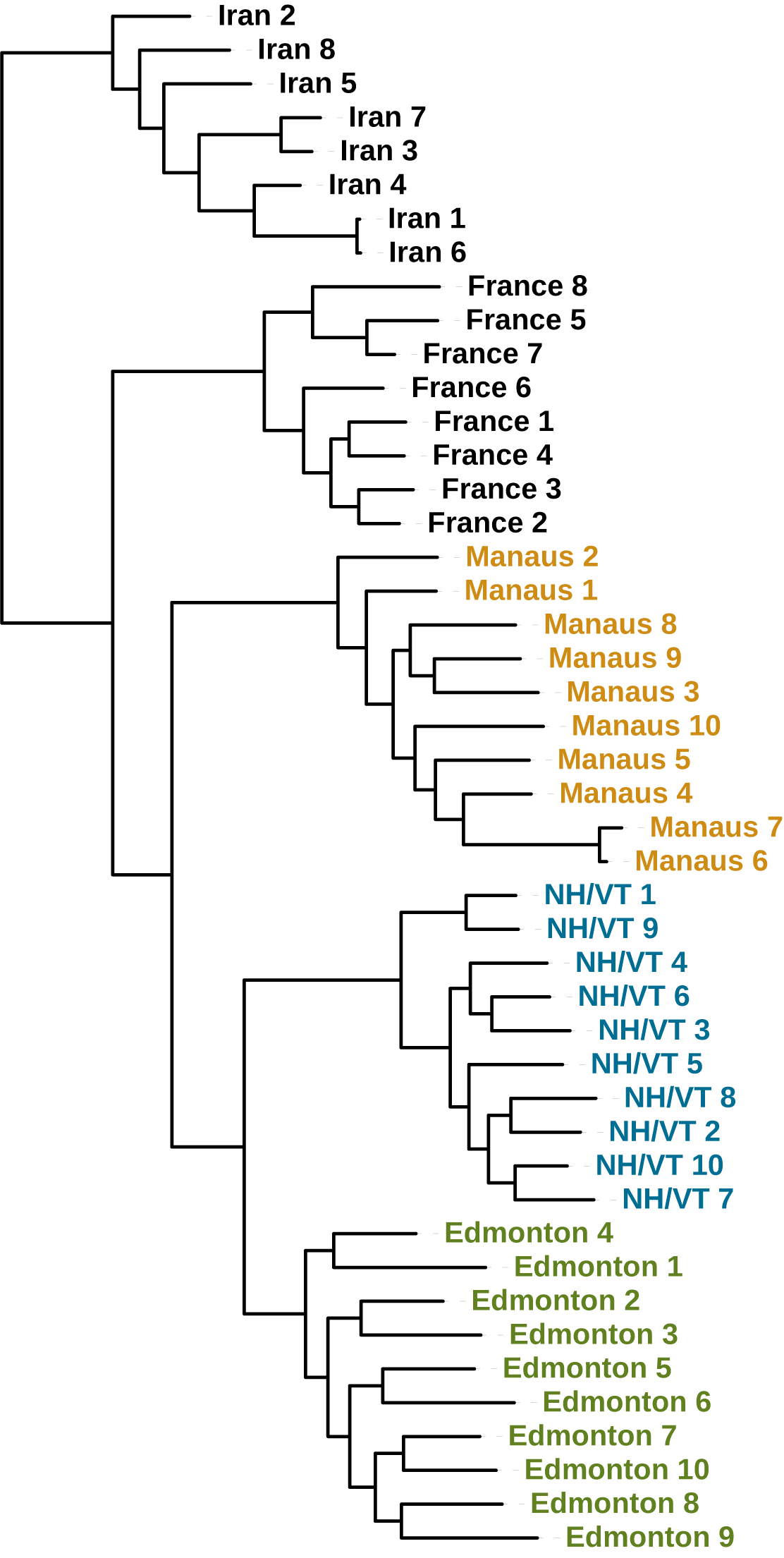

Supplement: S11 Fig — Maximum likelihood tree of focal house mouse populations from the ancestral range and North and South America. The tree is rooted using eight samples from Iran. (TIF) [file pgen.1010892.s012.tif]

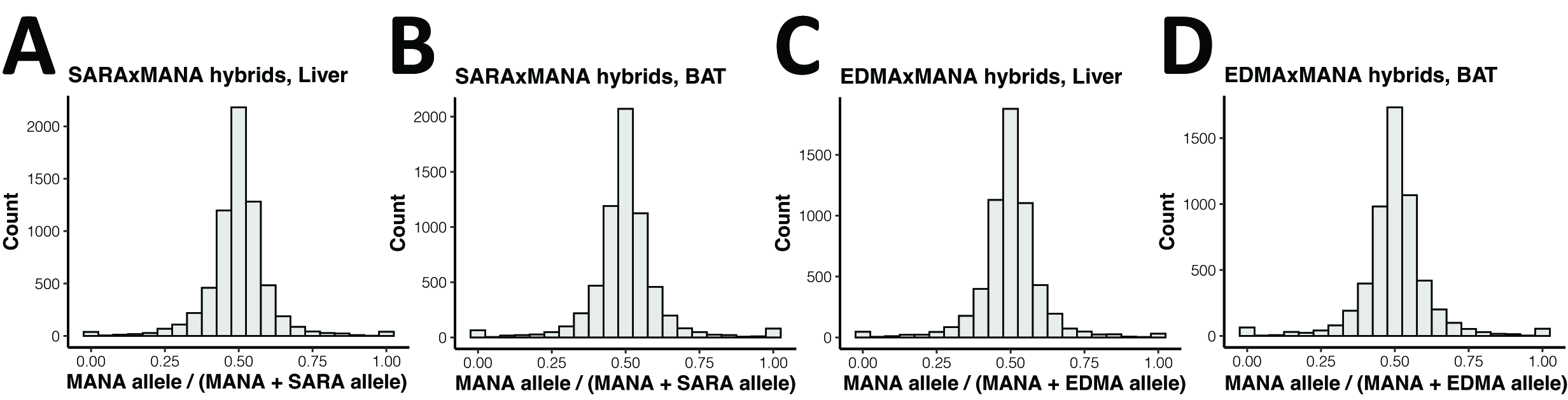

Supplement: S12 Fig — A-D). Ratio of F1 hybrid reads mapping to the MANA allele vs. all mapped reads is centered around 0.5 in each tissue for SARAxMANA (A-B) and EDMAxMANA (C-D) F1 hybrids, suggesting there is no mapping bias for reads preferentially mapping to the allele of one parent. (TIF) [file pgen.1010892.s013.tif]

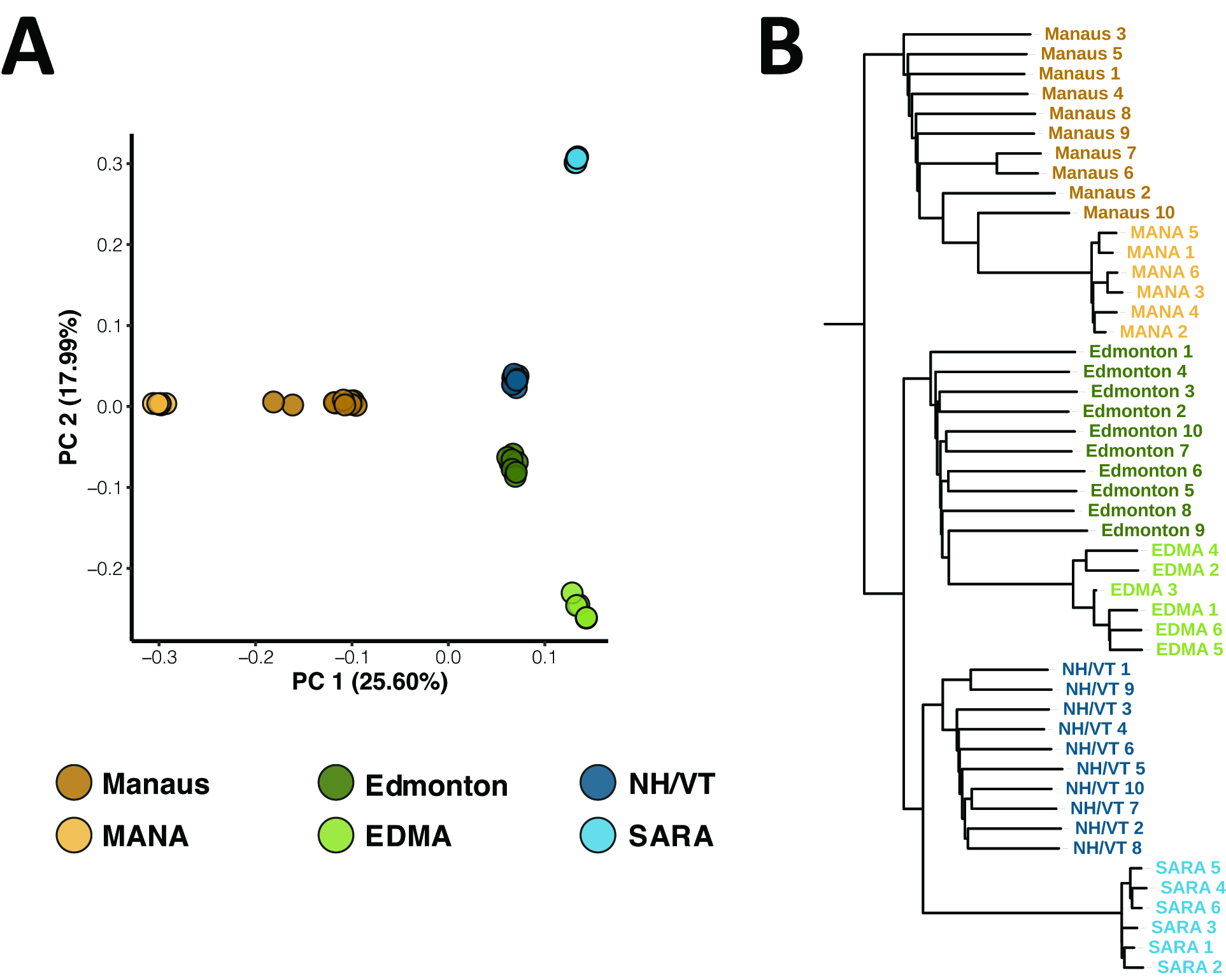

Supplement: S13 Fig — A). PCA of shared SNPs among focal population exome (Manaus, Edmonton, and NH/VT) and transcriptome (MANA, EDMA, and SARA) samples. B). Midpoint rooted neighbor joining tree of exome and transcriptome samples. (TIF) [file pgen.1010892.s014.tif]
