# Supplementary material for: Tissue-specific and cis-regulatory changes underlie parallel, adaptive gene expression evolution in house mice
Source: PLoS Genet. 2024 Feb 2;20(2):e1010892. doi: 10.1371/journal.pgen.1010892 (PMC10866503; doi:10.1371/journal.pgen.1010892)
Supplement: S1 Table — Enrichment of significant ASE genes in either liver or BAT among top 1% PBSn1 outliers in tests directly comparing NH/VT vs Manaus and Edmonton vs Manaus. Only PBSn1 outliers able to be tested for ASE and significant ASE genes represented in the relevant PBSn1 test are used. P-values represent percent of permuted distribution lying outside the observed overlap. Observed overlaps entirely outside the distributed are represented as p = ~0. (DOCX) [file pgen.1010892.s015.docx]

| PBS test | Top 1% outliers | Significant ASE | Overlap | p-value |
| --- | --- | --- | --- | --- |
| NH/VT focal – Manaus – Iran | 226 | 1102 | 60 | 0.0139 |
| Manaus focal – NH/VT – Iran | 176 | 1102 | 43 | 0.2528 |
| Edmonton focal – Manaus – Iran | 231 | 1001 | 81 | ~0 |
| Manaus focal – Edmonton – Iran | 196 | 1001 | 49 | 0.3046 |
