# Supplementary material for: Tissue-specific and cis-regulatory changes underlie parallel, adaptive gene expression evolution in house mice
Source: PLoS Genet. 2024 Feb 2;20(2):e1010892. doi: 10.1371/journal.pgen.1010892 (PMC10866503; doi:10.1371/journal.pgen.1010892)
Supplement: Attachment — Submitted filename: Durkin_etal_ResponseToReviewers.docx [file pgen.1010892.s029.docx]

**Reviewer #1:**

The study of house mice colonizing and adapting to the Americas from their ancestral range about 500 years ago continues to fascinate. This manuscript focuses on understanding the genetics underlying cold adaptation. By integrating expression data from mice reared under common laboratory conditions, in addition to controlled crosses allowing the assignment of cis vs trans regulatory changes, and genome wide SNP data analyses, the authors make a rather convincing point that adaptation to cold habitats involved mostly the same molecular changes in two widely separated populations (Saratoga Spring, New York and Edmonton, Alberta). They show that this evolution involves expression changes that are more likely to be cis regulatory, more likely to be tissue specific and more likely to show a signature of selection at the genomic level than expected by chance, and affect phenotypes such as body size, immune function and skeletal morphology.

I don’t have very much to criticize with data analyses, presentation and conclusions of this paper. As the authors state, finding the genetic basis of environmental adaptation is still a difficult task, and these data further our understanding significantly. My only point would be that the story is somewhat incomplete with respect to three aspects (detailed further below).

1) Terminology of “parallelism”

To me, the word parallelism implies that there is no room for the possibility that cold adapted alleles could have spread from one transect to the other by gene flow. In the earlier Plos Genetics paper frequently referred to the authors state: “An alternative possibility is that adaptation to high latitudes occurred once and that beneficial alleles were carried by rare migrants between the eastern and western transects. This seems less likely in light of the very recent colonization of house mice in the Americas and the well-supported phylogenetic relationships depicted in Figs 1B and S2”.

However, this possibility is completely ignored in the current paper. Long distance migration is common in house mice and it is now generally well known that just a trickle of gene flow is enough for adaptive introgression of alleles across populations, with many examples emerging. To me, it is still an open question to what degree the correlations observed in the data stem from true parallel evolution in situ rather than through some form of admixture, or a combination of both. A correlation is all that has been found so far. The authors themselves state: “Tissue specific and cis-regulatory changes underlie correlated adaptive gene expression evolution in house mice.” I feel that the word “correlation” or “correlated” would represent the current understanding of the system better than parallel or parallelism. Because the adaptive changes could result from introgression or independent selection from standing variation, I feel a better title would be to ignore these alternatives. The title therefore could read:

“Tissue-specific and cis-regulatory changes underlie adaptive gene expression evolution in house mice.”

This does not take away from the value of the paper, as similarities in both transects still speak strongly for adaptation being the driving force for the phenotypic and expression changes observed. And all other conclusions remain the same.

**This is a good point. We have added several sentences to the Introduction addressing this issue directly. The phylogeny of house mouse populations in North America suggests that mice invaded northern habitats twice, once in the east and once in the west. This is a separate issue from whether selection in the northern populations acted on new mutations, standing genetic variation, or alleles introduced by rare long-distance migrants. The absence of very long haplotypes adjacent to loci showing signatures of selection (from previous work which we cite) suggests that most selection did not act on new mutations, but we cannot distinguish between selection that acted on standing variation or selection that acted on alleles introduced by rare, long-distance migrants. We now acknowledge this clearly. But we still believe that this situation represents parallel evolution, since even if adaptive alleles were introduced into Edmonton from NY, they were independently pushed to high frequency by selection. (If, instead, cold-adapted NY mice colonized Edmonton en masse, this would be reflected in the phylogeny of the populations.) The situation is analogous to the parallel evolution seen in freshwater threespine stickleback fish, in which freshwater-adapted alleles are present in marine populations at low frequency and have been repeatedly pushed to high frequency by parallel evolution in similar freshwater habitats. We now mention this analogous situation in the text, hopefully to clarify our interpretation of the situation. This new text is on lines 117-128.**

2) Unique changes are not described

My second major point is somewhat related to the above and concerns the fact that in this paper no attempt is made to characterize the unique changes in more detail. Edmonton is 10 degrees further North with more extreme cold weather compared to Saratoga Spring. Unique changes are expected to contribute to adaptation to those differences in climate. I would be interested in knowing to what degree unique changes show similar characteristic than shared changes (i.e. are they also more likely to be cis and more likely to be tissue specific and more likely found in “increase growth genes” and more likely to show a signal of positive selection?). This information would give a somewhat more complete picture on just how similar both transects are. Of interest here is the fact that the Edmonton mice show a comparatively much larger proportion of expression changes in trans (Fig. 4E, 4F). In addition, there are actually more regulatory changes that are unique than shared in the Edmonton/Manaus comparison. I wonder if these results could be interpreted in such a way that adaptation to the more “benign” Saratoga Spring conditions are primarily in cis, but more extreme adaptations to even colder conditions require not only more changes but also more dramatic changes in trans?

**Thank you for this suggestion. We conducted an extensive new set of analyses comparing patterns between all DEGs, shared DEGs, and unique DEGs broken down by regulatory category, tissue specificity, and likelihood of being found in increased body size genes. We also compared enrichment for PBSn1 outliers in shared and unique significant ASE genes. We found no substantially different trends in shared and unique changes as compared to all expression changes generally. Although we agree that the slight differences in *trans*-regulation seen between SARA and EDMA are interesting, we don’t have enough data within the scope of this paper to make a meaningful conclusion from these trends. We detail our new results in the S1 Appendix and point readers to these results in the main text in lines 227-230.**

3) Selection pressures on southern habitats are not assessed.

Some of the expression differences observed in the comparisons between Manaus and the northern populations are expected to be due to adaptation of Manaus mice to warm habitats. The authors acknowledge this but make no attempt to estimate how much of the differences can be attributed to it, although early in the paper we are led to believe they will address the issue. I understand that this is a formidable challenge and as far as I can see can only be done indirectly, by looking at patterns of selection on the genomic level. So I am wondering why did the scan for selection not include the Manaus population? If the authors only intend to describe the genetic basis of cold adaptation, maybe it should be included in the title?

**This is an important point that was also addressed by Reviewer 2. We have conducted new selection scans that use exome data from Manaus both as a focal and outgroup population. Additionally, to have more power to detect *cis*-acting regulation, we changed all selection scan analyses to focus on genes with significant allele-specific expression (ASE), which is indicative of *cis*-regulation, instead of just *cis*-only genes. In scans where Manaus is the focal population with either France and Iran, Edmonton and Iran, or NH/VT and Iran as outgroups, we found no significant enrichment of *cis*-regulated genes among PBSn1 outliers (S9 Fig, S1 Table). This suggests that adaptive expression differences seen between northern mice and Manaus are more reflective of changes in New York and Alberta house mice than of Manaus house mice, and that adaptation of Manaus mice to warm habitats drives a smaller proportion of the expression divergence. These results are also consistent with the results of Ballinger et al. (2023) which we cite on line 333. For this reason, we still focus primarily on the selection scans with EDM and NH/VT as the focal populations and have added a discussion of our Manaus results to the main text (lines 317-333 and 447-456).**

Minor points:

Figure S11: I wonder if the authors could show branch length in the tree. This would give a better idea about overall divergence between the populations.

**Thank you for this suggestion. We changed the original neighbor joining tree to a maximum likelihood tree with branch lengths constructed using RAxML.**

Fig.2 lines should be reduced major axis or PCs or 95% ellipses not linear regression, as the slope of the line will depend on which data are plotted on the X or Y axis.

**We have changed all regression lines in plots comparing EDMA to SARA expression to major axis regression lines, which applied to multiple plots: Figures 2C, 3, S2C, S3, S5).**

Fig 4: P-values calculated using paired t-tests: what is the deg freedom

**Thank you for this question – we had erroneously reported these as paired t-tests, but they are actually Welch two-sample t-tests (Figure 4C-D, S4C-D). The data are independent, and therefore paired tests would not have been appropriate. We have corrected this in the main text and figure captions, and test statistics are given in lines 233-236.**

Page 22, line 484: should read …. 70-72F or 21-22C

**Thank you! We changed this to 70-72°F. Now line 541.**

**Reviewer #2:**

This paper examines parallel gene expression evolution in two replicate cold adapted populations of house mice. Using mice derived from two independent colonizations of cold environments (New York US, and Edmonton CA) the authors show that a large proportion of gene expression changes have evolved in parallel between the two cold-adapted populations, most of the parallel expression phenotypes are driven by cis-regulatory variants, and many of the parallel genes may bear signature of selection in cold environments (though there are caveats here - see below). In general, the paper focuses on an interesting and important set of questions, is exceptionally well-written, and the analyses seem technically sound and well-executed (for the most part). It’s an interesting paper, and I enjoyed reading it.

My only major critiques are associated with the genomic selection scans, which seemed a bit clunky. As a general critique, there is a lack of overlap between the populations used in the transcriptomic analyses and those used in the genome scans. While the experiments used to identify gene expression evolution were focused on mice from New York, Edmonton, and Brazil, the populations used in the genome scans, varied depending on the comparison. However, there was the only direct overlap for mice from Edmonton (and even here it is not clear if the same locality was sampled). Mice sampled from Vermont and New Hampshire served as a proxy for the New York mice, and there was no analysis of genetic variation in mice from warm-adapted Brazilian population. Since mice mice from New York and Brazil, were used to identify morphological and regulatory phenotypes associated with cold adaptation, it seems odd to exclude them from the analyses designed to detect genomic signatures of selection.

The reasons for this omission stem largely from the fact that genome-scans involved a reanalysis of previously published data - there were no population genomic data generated in this study (to my understanding anyway). While I understand and appreciate the value of reanalyzing existing datasets, in this case, the approach does not seem ideal. The genome scans relied on the population-branch statistic, which of course, requires a three-way comparison allele frequency variation. Because the transcriptomic analysis involved two cold-adapted populations and one warm-adapted population, it was not possible to identify loci that specifically bear signatures of cold-adaptation using a PBS analysis of those three focal populations, and the authors recognized this problem. Thus, by necessity, the authors needed to sample additional outgroups. It seems that the best design for these comparisons, however, would have involved a cold-adapted population (NY or ED), Brazil, and an appropriate warm-adapted outgroup, repeating the analysis for each focal cold adapted population. This design could replace the authors' 'nested analysis' and the outliers in these repeated PBS analysis could more cleanly be interpreted as loci under parallel selection in each of the cold-adapted populations and related back to the gene expression patterns.

The authors did something like this, but the outgroups varied by comparison, proxy samples were used for one cold-adapted population (i.e. NH/VT to represent NY), and the focal warm-adapted population was never examined. These issues make relating the results of the genome scans to the patterns gene expression evolution a bit murky. The authors do address some of these issues (e.g. lines 295-301), but much of that discussion focuses on justification of Eurasian mice as outgroups. This is an important consideration, but at least equally important is whether VT/NH is an appropriate proxy for NY. The sole justification is that these locations are “geographically close” (line 295). Is there any appreciable population structure between these two localities? Do VT/NH mice have similar body sizes and limb lengths as the NY mice? These details could and should be easily added to better justify the choice. Similarly, why weren’t genomic data from Brazilian mice included? Are they not publicly available, or is another, better-grounded reason?

Similarly, the data from both the Edmonton and VT/NH mice were exome data (line 296). This seems an odd choice for a paper aimed understanding regulatory evolution. How confident are the authors that this strategy captures variation in and selection on relevant cis-regulatory elements? Details on exome capture design, like whether and how much flanking/non-exon sequence was captured would be useful, but again, it begs the question of whether the publicly available data are well-suited to address the questions at hand.

All of these issues could have been avoided if population genomic data (e.g. low coverage whole genome data) had been gathered for the three focal populations here. That said, I am certainly sensitive to financial and other constraints that often preclude optimal study design.

**Thank you for this thoughtful and thorough comment. We have undertaken several new analyses to address these points. We have broken down our response into three discrete categories below.**

**1. No analysis of Manaus at the genomic level:**

**We have conducted a number of new selection scans that use exome data from Manaus both as a focal and outgroup population. Additionally, to have more power to detect *cis*-acting regulation, we changed all selection scan analyses to focus on genes with significant allele-specific expression (ASE), which is indicative of *cis*-regulation, instead of just *cis*-only genes.**

**In scans where Manaus is the focal population with France and Iran as outgroups, we found no significant enrichment of *cis*-regulated genes among PBSn1 outliers (S9 Fig). Additionally, we conducted selection scans like those suggested by reviewer that directly compared Manaus and the Northern populations, with Iran as an outgroup. Iran is ancestral to house mice in the Americas and is a warm climate. When Manaus is the focal population in these scans, we also found no significant enrichment of *cis*-regulated genes among PBSn1 outliers (S1 Table), however we did see significant enrichment when either northern population was the focal group. This suggests that adaptive expression differences seen between northern mice and Manaus are more reflective of changes in New York and Alberta house mice, and that adaptation of Manaus mice to warm habitats drives a smaller proportion of the expression divergence. For this reason, we still focus primarily on the selection scans with EDM and NH/VT as the focal populations, and have added a discussion of our Manaus results to the main text (lines 317-333 and 447-456).**

**2. New Hampshire/Vermont exome data as a proxy for SARA expression data:	Unfortunately, we do not have appropriate morphological data to compare Saratoga Springs, NY mice to New Hampshire/Vermont mice (i.e., we only have data from lab reared NY mice and wild caught NH/VT mice). To address the use of NH/VT mice, we assessed genetic similarity in the exome and transcriptome data using PCA and phylogenetic analyses. We detail these results in the S1 Appendix and point readers to these results in lines 313-315. We found the relationship between SARA and NH/VT mice to be similar to the relationship between EDMA and EDM mice or the relationship between MANA and MAN mice.**

**3. The use of exome data to speak to regulatory evolution:**

**We have added a discussion of the use of exome data to speak to regulatory evolution to the methods section (lines 741-746): “Details on exome capture design can be found in Phifer-Rixey et al. [20]. SNPs identified using exome probes are in exons, but a large proportion are also in adjacent intronic and inter-genic regions [20]. Because linkage disequilibrium (LD) in wild house populations extends for 10’s of kb [63], SNPs in exome data are expected to be frequently linked to regulatory SNPs, making these data suitable for investigating regulatory evolution.”**

Below are some more minor points for the authors to consider:

Line 267-268: If space constraints are not too stringent, it would be better to present the BAT results in the main text rather than relegating them to the supplement.

**We agree that it would be nice to highlight both tissues in the main text. However, results in BAT were comparable to those in liver and there are four supplementary figures of BAT data, which would increase our main text figure number to ten. To not present an overwhelming amount of data in the main text, we opted to keep the BAT figures in the supplement.**

Lines 455-458: This is a fine suggestion, but I think it is important to remind the readers that co-expression networks (e.g. WGCNA) are correlation networks and not true interaction networks, and as such, are not a direct measure of the extent of pleiotropy.

**Thank you for this comment. We have changed this part of the manuscript to read: “The distinction between *cis*- and *trans*- changes in our study does not allow us to assess “degrees” of pleiotropy, but future studies using eQTL mapping or gene co-expression networks, which can act as a proxy for pleiotropy, could be used to ask whether genes with intermediate levels of connectedness are most likely to underlie parallel evolution.” (lines 508-511).**

Lines 460-481: This section seems a little premature/overstated since no direct phenotype-genotype connection has been made here. I do agree that this approaches such as those employed here are a good way to nominate candidate genes for further analysis though. Consider sharpening the focus a bit more, and make clearer that variation (neither expression nor allelic) has not been directly tied to adaptive phenotypes in this study. That distinction might not be clear to the casual reader in its current state.

**We have revised this section to highlight that our findings are only an initial step in identifying candidate genes underlying complex traits. We removed the word “strong” from the section title (line 513), and we refer to our approach as a first step in identifying candidate genes, and highlight making direct links between phenotype and genotype as the next step (lines 527-530).**

**Have all data underlying the figures and results presented in the manuscript been provided?**

Large-scale datasets should be made available via a public repository as described in the *PLOS Genetics* [data availability policy](http://journals.plos.org/plosgenetics/s/data-availability), and numerical data that underlies graphs or summary statistics should be provided in spreadsheet form as supporting information.

Reviewer #1: Yes

Reviewer #2: **No:** It seems as though the RNASeq have not yet been submitted to the NCBI SRA. The cover letter includes a place holder number for the BioProject ID.

**The RNAseq data generated here have been submitted to the NCBI SRA under the BioProject ID PRJNA1009445.**
